# Editorial: Scalable Bioinformatics: Methods, Software Tools, and Hardware Architectures

**DOI:** 10.3389/fgene.2021.822986

**Published:** 2022-01-05

**Authors:** Nikolaos Alachiotis, Tze Meng Low, Pavlos Pavlidis

**Affiliations:** ^1^ Faculty of EEMCS, University of Twente, Enschede, Netherlands; ^2^ Department of Electrical and Computer Engineering, Carnegie Mellon University, Pittsburgh, PA, United States; ^3^ Foundation for Research and Technology-Hellas, Heraklion, Greece

**Keywords:** autism spectrum disorder, basic local-alignment search tool, BLAST, SMUFIN, copy number variations, SARS–CoV–2

Advances in DNA sequencing technology have contributed to the accumulation of molecular sequence data at an unprecedented pace, since whole genomes can now be sequenced rapidly, accurately, and cost effectively. When methods and tools are not specifically designed to handle big volumes of data efficiently, large-scale analyses practically become infeasible due to the explosion in processing and memory requirements. Bioinformatics algorithms frequently rely on approximations and heuristics to yield computationally tractable implementations, at the cost of performing less thorough analyses. This Research Topic presents a series of works that connect computational problems in the fields of Bioinformatics and Computational Biology with software and hardware solutions from the fields of Computer Science and Computer Engineering to address scalability issues across a variety of Bioinformatics problems.

The Basic Local-Alignment Search Tool, BLAST (Altschul et al., 1990), is one of the most widely used algorithms to search for sequence similarities in Bioinformatics. Gálvez et al. introduce BLVector, a heuristic algorithm that adapts high-level concepts of BLAST+ to many-core x86 architectures with Single-Instruction Multiple Data (SIMD) vector instructions of the Advanced Vector eXtensions (AVX)-512 instruction set. BLVector outperforms BLAST+ for mid-size protein sequences (∼750 amino acids), and retrieves a much larger set of results than BLAST+ when applied to longer proteins, at the cost of a longer execution time. BLVector can be up to an order of magnitude faster than BLAST+ on various many-core processor architectures, and the authors suggest that BLVector and BLAST+ can be considered as complementary tools.

Autism spectrum disorder (ASD) is a neurodevelopmental disorder that has been extensively studied over the past decades (Cox et al., 1999; Marshall et al., 2008; Ozonoff and Iosif, 2019).

Garbulowski et al. developed an analysis pipeline to construct interpretable machine learning models and performed an analysis of multiple cohorts of control-case studies of Autism Spectrum Disorder (ASD). The analysis revealed that autism is the most severe subtype of ASD, while pervasive developmental disorder-not otherwise specified (PDD-NOS) and Asperger syndrome are closely related and milder subtypes of ASD. Additionally, the authors analyzed the most important ASD-related features described in terms of gene co-predictors, finding a strong co-predictive mechanism and possible co-regulation between genes *EMC4* and *TMEM30A*. This study showcases one more application of Machine Learning and outlines its potential in providing insights into important medical and biological questions while encouraging the deployment of other techniques such as deep learning (LeCun et al., 2015) as well.

Analyzing a patient’s genetic data is the first step toward precision medicine. Cadenelli et al. present SMUFIN-F, a memory-efficient approach to perform mutation detection on commodity personal computers with a small amount of DRAM. The analysis of somatic mutations is essential to the study and treatment of cancer. The authors modified SMUFIN (Moncunill et al., 2014; Cadenelli et al., 2017), the leading algorithm for detecting somatic mutations, to operate on big data structures that reside in secondary storage instead of DRAM. The authors show that when SMUFIN-F allocates 16 times less memory than SMUFIN, for the same problem size, it is only 1.24x slower than SMUFIN. Based on the observation that two commodity PCs running SMUFIN-F deliver the same throughput as an enterprise server running SMUFIN, the authors estimate that SMUFIN-F achieves the same performance at only 36% of the capital cost and 45% of the operational cost (energy).

The exponential growth of genome sequences available has spurred research on pattern detection with the aim to extract evolutionary signals. Pechlivanis et al. describe a novel computational framework for identifying potentially meaningful features based on *k*-mers retrieved from unaligned sequence data. The framework employs unsupervised machine learning to identify characteristic *k*-mers of the input dataset across a range of different *k*-values and within a reasonable time frame. The authors applied their approach on 8,693 SARS-CoV-2 genomes and identified *k*-mers at the nucleotide level, from which they constructed an evolutionary tree. Furthermore, integration with population demographic and chronological metadata led to the identification of unique clusters and time correlated features among the available sequences and *k*-mers. The results can be beneficial for a better understanding of the genetic diversity of SARS-CoV-2.

Copy number variations (CNVs) (Freeman et al., 2006; Redon et al., 2006) are important genomic structural variations that are widespread in the human genome and cause a variety of complex diseases. Huang et al. developed an improved method called CNV-MEANN (CNV detection of neural network based on mind evolutionary algorithm). CNV-MEANN introduces a new feature called mapping quality to evaluate whether the mapping position can be trusted. In addition, it considers the influence of the loss categories of CNV on disease prediction and deploys a mind evolutionary algorithm to optimize the backpropagation (neural network) neural network model while calculating individual scores for each genome bin to predict CNVs. CNV-MEAN was tested with both simulated and real datasets, and outperformed other methods with respect to sensitivity, precision, and F1-score. Furthermore, CNV-MEAN was able to detect CNVs that were not detected by other approaches.

Scalable solutions and high performance are imperative in the fields of Bioinformatics, and the COVID-19 pandemic brought Bioinformatics into the spotlight, revealing that several existing methods, algorithms, and tools were not well prepared to handle large amounts of genomic data efficiently. Performant and memory-aware solutions are required to ensure that future computing systems will be able to keep up with the molecular data avalanche. This Research Topic highlights the need for an interdisciplinary approach to address scalability issues in Bioinformatics with the hope of paving the way for fruitful future collaborations between researchers from these fields.
